# Mathematical Description of the Increase in Selectivity of an Anion-Exchange Membrane Due to Its Modification with a Perfluorosulfonated Ionomer

**DOI:** 10.3390/ijms23042238

**Published:** 2022-02-17

**Authors:** Anton Kozmai, Natalia Pismenskaya, Victor Nikonenko

**Affiliations:** Membrane Institute, Kuban State University, 149, Stavropolskaya Str., 350040 Krasnodar, Russia; kozmay@yandex.ru (A.K.); n_pismen@mail.ru (N.P.)

**Keywords:** ion-exchange membrane, electric conductivity, diffusion permeability, permselectivity, structure–properties relationship, modification, microheterogeneous model

## Abstract

In this paper, we simulate the changes in the structure and transport properties of an anion-exchange membrane (CJMA-7, Hefei Chemjoy Polymer Materials Co. Ltd., China) caused by its modification with a perfluorosulfonated ionomer (PFSI). The modification was made in several stages and included keeping the membrane at a low temperature, applying a PFSI solution on its surface, and, subsequently, drying it at an elevated temperature. We applied the known microheterogeneous model with some new amendments to simulate each stage of the membrane modification. It has been shown that the PFSI film formed on the membrane-substrate does not affect significantly its properties due to the small thickness of the film (≈4 µm) and similar properties of the film and substrate. The main effect is caused by the fact that PFSI material “clogs” the macropores of the CJMA-7 membrane, thereby, blocking the transport of coions through the membrane. In this case, the membrane microporous gel phase, which exhibits a high selectivity to counterions, remains the primary pathway for both counterions and coions. Due to the above modification of the CJMA-7 membrane, the coion (Na^+^) transport number in the membrane equilibrated with 1 M NaCl solution decreased from 0.11 to 0.03. Thus, the modified membrane became comparable in its transport characteristics with more expensive IEMs available on the market.

## 1. Introduction

The selectivity of ion-exchange membranes (IEM) in relation to counterion transport is the main functional property of this type of membranes. This property determines the possibility of desalination and concentration of electrolyte solutions by the electrodialysis method [1,2,3]. A good counterion permselectivity is needed for numerous applications of IEMs: for processing solutions in the agro-food industry, in particular, for the production of fertilizers [4,5], in fuel cells [6,7,8,9], flow batteries [9,10,11] and other processes [12,13,14,15]. Water treatment remains an important application of IEMs [16,17], the significance of which increases due to the degradation of water quality in natural sources affected by human activity [18,19].

Commercial IEMs, depending on the manufacturing method and structure, are divided into two large groups: homogeneous and heterogeneous membranes. The competition between these two types of IEM has lasted for more than half a century [20,21]. In general, the electrochemical properties of homogeneous membranes, including the selectivity to counterion transport, are superior to those of heterogeneous membranes, the main advantage of which is low production cost and higher stability during operation.

The technology of IEM production is constantly being improved. However, the development of new types of membranes is rather costly; therefore, despite their diversity, their number remains limited [4]. Modification of membranes is carried out using a small amount of modifying agents and, usually, leads to a slight increase in their cost [22].

The modification of membranes is widely used in membrane technology. The reverse osmosis method became widely used only after the illustrious innovation of Loeb and Surirajan, who, in the early 1960s, proposed to produce membranes with a thin active surface layer [23]. As for IEMs, the use of membranes consisting of a substrate and an active layer is less common, if bipolar membranes are not taken into account. Apparently, some of the first to use such a technique were Kedem et al. [24]. They covered heterogeneous IEM with a thin layer of a homogeneous ion-conducting film to “suppress concentration polarization”. As a result, the limiting current density increased, and the potential drop across the membrane at a given current density decreased. A similar technique was used in Refs. [25,26]. In addition to the above effects, membranes with a homogenized surface showed a significant increase in fouling resistance: the growth rate of precipitation on the membrane surface in solutions containing hardness ions significantly decreased or was completely absent [27].

A thin active layer on an IEM is also used to increase the specific selectivity of ion transport across a composite membrane with respect to singly charged ions [28,29,30] or with respect to nitrates as compared to chlorides [31]. The mechanisms of the modifying layer formation in the process of multilayer membranes manufacturing, as well as the reasons for the selectivity of these membranes to individual ions are considered in Refs. [28,29]. However, more often, an empirical approach is used, where the modifying layer nature and thickness are determined by the trial-and-error method [32,33]. The deposition on the membrane surface of a thin layer with fixed ions charged opposite to the charge of the substrate membrane makes it possible to obtain asymmetric bipolar membranes. By adjusting the fixed ions density and their nature in a thin layer, as well as the thickness of this layer, it is possible to control the rate of H^+^ and OH^−^ ions generation at the bipolar boundary, while retaining the function of the membrane for desalination and concentration of electrolytes [34,35,36].

Although there are numerous papers devoted to the development of monovalent-ion-selective membranes, including the method of modification [12,37,38,39,40,41,42,43], there are only a few papers where this method is used to improve the counterion permselectivity [44,45,46]. Sarapulova et al. [44], in order to improve the counterion permselectivity, relied on a known fact that coions mainly pass through the membrane within relatively large (macro)pores [44,47,48]. Therefore, the idea of their modification was to clog the macropores with a homogeneous microporous ion-exchange material. After the modification of a homogeneous CJMA-7 membrane, the coion transport number decreased by 2.5 times (in 1.0 M NaCl solution). This interesting and promising result stimulates theoretical research to better understand the effect and to assess the prospects of the applied approach.

To describe the effect of clogging the macropores, the applied model must take into account the ion transport in the pores of different size—not only in the macropores, but also in micro- and mesopores. In the literature, two main types of models, which are applied to describe the ion and water transport in IEM, can be distinguished: (1) “solution-diffusion” [49,50,51,52] and (2) “pore-flow” models [53,54]. Models of the first type do not allow one to determine an explicit relationship between the parameters of the membrane structure and transport coefficients. However, this relationship can be established, when considering the membrane as a multiphase microheterogeneous system (effective-medium approach) [55]. Models of the second type simulate the ion and water transport directly in a membrane pore. However, the complexity of the mathematical description allows for considering only pores of one specific size.

The description of ion transport in a membrane as an effective porous medium was undertaken by Mackie and Meares [56], Prager [57] and others. The membrane is represented as a structure containing a non-conductive backbone and a system of pores. They introduced the tortuosity factor; in addition, several different equations for calculation of this parameter as a function of the volume fraction occupied by the polymer matrix impermeable to ion diffusion were proposed. Gluckauf [58] took into account the fact that the membrane contains pores of different size, introducing the function of pore distribution as a function of their radii. In his approach, diffusion permeability is considered as an average value for a medium, in which the local value of the permeability coefficient is continuously changing in space. However, in the considered models, only the diffusion process is studied, but electromigration is not taken into account.

The microheterogeneous (MH) model [59] takes into account the presence of pores of different sizes. The membrane parts containing micropores form a gel phase considered as a homogeneous medium. The elements of the gel phase are separated from each other by intergel spaces. These intergel spaces are filled with the electrically neutral solution being in equilibrium with a local fragment of the membrane. They are interpreted as macropores and central parts of mesopores outside the electrical double layer on the pore walls. In the MH model, both the diffusion and electromigration are taken into account. Application of the MH model for a large number of homogeneous and heterogeneous membranes has shown that it adequately describes the concentration dependencies of conductivity, diffusion permeability and transport numbers [52]. The analysis made above shows that, for describing the effects caused by the modification of ion-exchange membranes, carried out in Ref. [44], the application of the microheterogeneous model seems the most appropriate.

In our work, we applied this model to study such effects, i.e., the influence of ion exchange membrane modification [44] on its conductivity, diffusion permeability and ion transport numbers. While the main idea of this modification was to clog the macropores, other treatments that positively affect the membrane permselectivity were performed. We will describe the responses of the membrane structure to these treatments through changes in the parameters of the microheterogeneous model. For the first time, we take into account that the intergel spaces are not completely filled with the aqueous solution, but partially with the PFSI material.

## 2. Results and Discussion

### 2.1. Model Formulation

The microheterogeneous model (MH) [59] provides a rather simple description of membrane transport characteristics as functions of a single set of six parameters, which include two thermodynamic parameters (the ion-exchange capacity of the gel phase ($\bar{Q}$) and the Donnan equilibrium constant ($K_{D}$); two structural parameters (the volume fractions of the gel phase (*f*_1_) and intergel spaces (*f*_2_, *f*_1_ + *f*_2_ = 1), and a parameter (*α*), which reflects the relative disposition of the gel and solution phases); and two kinetic parameters (the ion diffusion coefficients (${\bar{D}}_{i}$) in the gel phase, *i* = “+” and “−” for the cation (Na^+^ ion) and anion (Cl^−^ ion), respectively). In addition, the intergel solution is also characterized by ion diffusion coefficients ($D_{i}$). In its conventional version, the model assumes that the intergel solution is identical to the external equilibrium solution, hence, $D_{i}$ do not differ from the ion diffusion coefficients in free solution. Parameter *α* can change between −1 and +1: *α* = −1 refers to the case where the elements of the gel and solution phases are connected in series; *α* = +1 refers to the case where these elements are connected in parallel.

The membrane conductivity ($\kappa^{*}$), diffusion permeability ($P^{*}$) and ion transport numbers ($t_{i}^{*}$) in the membrane can be calculated through the effective membrane conductance coefficients, $L_{i}^{*}$ [59]:

(1) $$\kappa^{*}=\left(z_{+}^{2}L_{+}^{*}+z_{-}^{2}L_{-}^{*}\right)F^{2}$$

(2) $$P^{*}=RT\left(z_{+}L_{+}^{*}t_{-}^{*}+|z_{-}|L_{-}^{*}t_{+}^{*}\right)/c$$

(3) $$t_{i}^{*}=z_{i}^{2}L_{i}^{*}/\left(z_{+}^{2}L_{+}^{*}+z_{-}^{2}L_{-}^{*}\right)=z_{i}^{2}L_{i}^{*}F^{2}/\kappa^{*},i=+,-$$

where $c=\left|z_{i}\right|c_{i}$ is the equivalent electrolyte concentration in the intergel solution (expressed in eq L^−1^). Expressions (1) and (2) are deduced from the irreversible thermodynamics using also the effective medium theory approach [60].

According to the MH model, the effective coefficients $L_{i}^{*}$ in the membrane are found through the ion conductance coefficients in the individual constituent phases:

(4) Li*=f1L¯iα+f2Liα1α

where ${\bar{L}}_{i}$ refers to the gel phase, and $L_{i}$, to the intergel electroneutral solution.

When describing the transport in the gel phase, we apply the assumptions of the Teorell–Meyer–Sievers (TMS) model. The gel phase is considered as homogeneous, the Nernst–Planck equation and the local electroneutrality condition are used there as well as in the intergel solution. $L_{i}$ and ${\bar{L}}_{i}$ are expressed as functions of the ionic diffusion coefficients, $D_{i}$ and ${\bar{D}}_{i}$, and the concentrations, $c_{i}$ and ${\bar{c}}_{i}$ in the corresponding phase [59]:

(5) $$L_{i}=D_{i}c_{i}/RT,\;{\bar{L}}_{i}={\bar{D}}_{i}{\bar{c}}_{i}/RT$$

Since, locally, the gel phase and intergel solution are assumed to be in equilibrium, concentrations $c_{i}$ and ${\bar{c}}_{i}$ in these phases are linked by the Donnan relation:

(6) $$\frac{{\left({\bar{c}}_{-}\right)}^{1/z_{-}}}{{\left({\bar{c}}_{+}\right)}^{1/z_{+}}}=K_{D}\frac{{\left(c_{-}\right)}^{1/z_{-}}}{{\left(c_{+}\right)}^{1/z_{+}}}\;\mathrm{for}\;\mathrm{an}\;\mathrm{AEM},$$

(7) $$\frac{{\left({\bar{c}}_{+}\right)}^{1/z_{+}}}{{\left({\bar{c}}_{-}\right)}^{1/z_{-}}}=K_{D}\frac{{\left(c_{+}\right)}^{1/z_{+}}}{{\left(c_{-}\right)}^{1/z_{-}}}\;\mathrm{for}\;a\;\mathrm{cation}\text{-}\mathrm{exchange}\;\mathrm{membrane}\;\mathrm{or}\;\mathrm{PFSI}\;\mathrm{material}$$

The concentration of coions in the gel phase can be found when solving Equation (6) together with the electroneutrality condition in the gel phase, Equation (8):

(8) $$z_{-}{\bar{c}}_{-}+z_{+}{\bar{c}}_{+}=\bar{Q}$$

The exchange capacity of the gel phase ($\bar{Q}$) is linked to the ion-exchange capacity of the membrane (Q) as follows: $\bar{Q}=Q/f_{1}$.

### 2.2. Comparison of Simulation and Experiment

**Figure 1:**
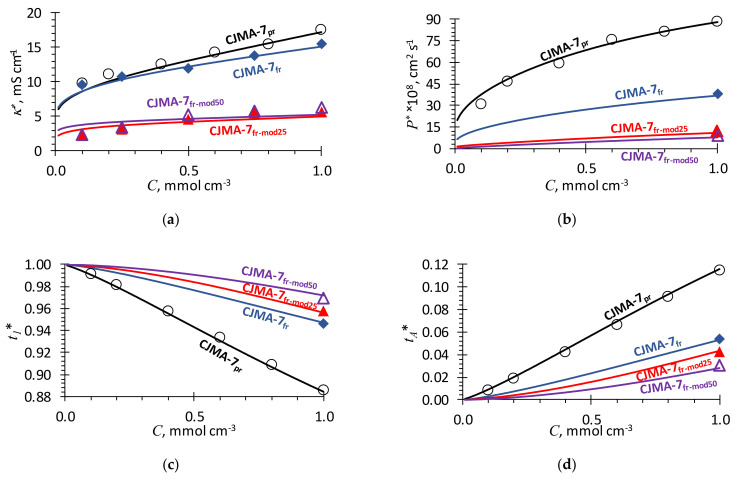
Experimental and simulated using the MH model concentration dependencies of (**a**) conductivity, (**b**) diffusion permeability, (**c**) counterion and (**d**) coion transport numbers for studied membrane samples (indicated near the corresponding curve). Markers denote the experimental data, lines denote the results of the simulation.

Figure 1 shows the experimental and simulated concentration dependencies of conductivity, diffusion permeability as well as counterion and coion transport numbers for the membranes under study. As it can be seen, there is a good agreement, when the MH model parameters presented in Table 1 are used. Each stage of the membrane processing is reflected by a change in the parameters. Below, we discuss the variations in the membrane structure occurring due to the treatment of the pristine membrane at each stage of its modification. The corresponding changes in the model parameters are also considered.

### 2.3. Exposure to a Low Temperature

As it is described in detail in Section 3.2, a sample of the pristine membrane, CJMA-7_pr_, was kept in a fridge at 4–6 °C for 12 months. Figure 1a shows that the conductivity of CJMA-7_fr_ decreased up to 12% as compared to the conductivity of CJMA-7_pr_; the conductivity changed more strongly in the range of high concentrations. With that, the diffusion permeability decreased by 57% at $C_{s}$ = 1 mol L^−1^, the only concentration at which the measurement of $P^{*}$ was performed in Ref. [44]. Since, in the first approximation, the coion transport number, $t_{A}^{*}$, is proportional to $P^{*}/\kappa^{*}$ (see Equation (12)), a stronger decrease in $P^{*}$ as compared to a decrease in $\kappa^{*}$ results in a significant decrease in $t_{A}^{*}$.

**Figure 2:**
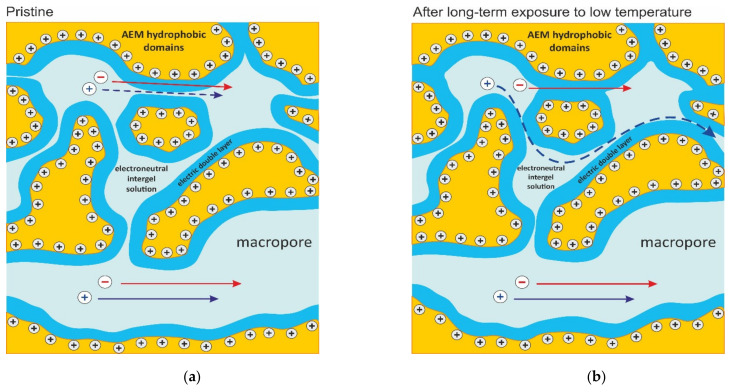

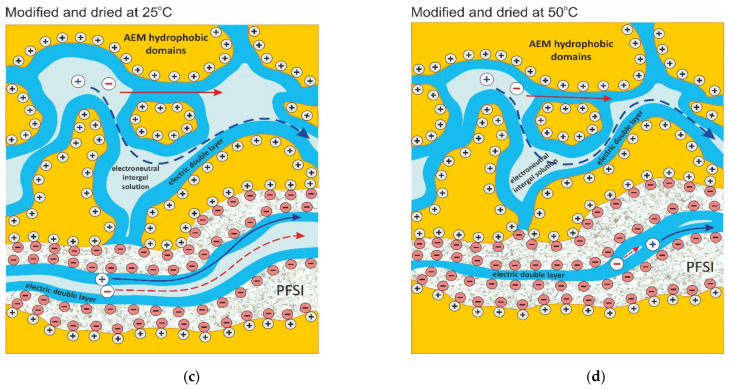
Schematic representation of the changes in studied membrane structure at different stages of its treatment: (**a**) CJMA-7_pr_, (**b**) CJMA-7_fr_, (**c**) CJMA-7_fr-mod25_ and (**d**) CJMA-7_fr-mod50_. The membrane structure is presented in accordance with modern concepts [65,66,67]: mesopores (ion clusters) are connected to each other by narrower ion-conducting channels; the macropores, which penetrate these finely porous areas, are due to structural defects, including the gaps between ion-exchange material and non-conductive fillers, such as fibers of reinforcing cloth [48]. The arrows show ion pathways during salt diffusion through the membrane. An animation that combines (**a**–**d**) is presented in Supplementary Materials.

The fact that the diffusion permeability decreased when the membrane was maintained for a long period at low temperature could be explained by the reorientation/relaxation of the chains of the weakly cross-linked polymer forming the membrane matrix. According to Refs. [61,62,63], such a reorientation/relaxation at low temperatures leads to a more compact packing of polymer chains, which results in a narrowing of the pores (Figure 2b). With decreasing temperature, the polymer structure becomes less loose, the free volume decreases [63]. Note, that the relaxation of an ion exchanger’s polymer matrix during exposure to a low temperature takes quite a long time; for example, in the case of Nafion material, this time exceeds 1000 h [64]. In the terms of the MH membrane parameters, these changes in the membrane structure under a low temperature can be interpreted as an increase in the volume fraction of the microporous gel phase, *f*_1_, and a corresponding decrease in the volume fraction *f*_2_ of intergel spaces (including the electroneutral solution in the central part of macro- and mesopores). This change in the values of the *f*_1_ and *f*_2_ parameters when converting CJMA-7_pr_ to CJMA-7_fr_ is presented in Table 1. Another result of the narrowing of pores (namely of the micropores) is a decrease in the ionic diffusion coefficients in the gel phase. Recall that the diffusion coefficients in the electroneutral solution located in the macro- and mesopores are assumed the same as in the free solution. However, decreasing space in the micropores impedes the ion mobility. Moreover, the diffusion coefficient of coion should decrease more than that of counterion (Table 2). This is explained by the fact that a reduction in the distance between opposite pore walls leads to a decrease in the thickness of the electrically neutral or weakly charged solution located between two electrical double layers (EDLs) adjacent to the pore walls. This weakly charged solution is a pathway for coions through a narrow channel connecting two relatively large pores (ion clusters) (Figure 2). The EDLs are almost impermeable to coions because of the effect of Donnan coion exclusion: repelling of coions by the electrostatic force acting between them and the fixed positively charged functional groups [1]. The narrowing of micropores makes some of them completely impermeable to coions and, thus, they must get around some regions of the gel phase. Therefore, the resulting pathway for coions became longer and more tortuous: compare Figure 2a,b. Since the MH model does not apply the tortuosity factor, we take into account the longer pathway of coions in the gel phase of CJMA-7_fr_ by decreasing the diffusion coefficient of the coion (${\bar{D}}_{+}$) to a greater extent than that of the counterion (${\bar{D}}_{-}$) (Table 1).

Due to a more compact structure, the concentration of fixed functional groups in the gel phase, $\bar{Q}$, slightly increases when passing from CJMA-7_pr_ to CJMA-7_fr_, and the Donnan constant, $K_{D}$, decreases (Table 1). The latter means a stronger exclusion of coions from the gel phase. Since some domains of the gel phase becomes non-permeable for coions, the contribution of the ion transport in parallel ways through gel phase and intergel solution increases, hence, the value of the *α* parameter decreases.

### 2.4. Effect of Membrane Modification with PFSI Solution

Modification of CJMA-7_fr_ membrane with PFSI (see CJMA-7_fr-mod25_ or CJMA-7_fr-mod50_ in Figure 1) results in a dramatic decrease (almost by 60%) in the conductivity as compared to CJMA-7_fr_. At the same time, the diffusion permeability of the modified samples decreased even more significantly (up to 75%), which led, according to Equation (12), to a noticeable decrease in the coion transport numbers: by about 20% in the case of drying at 25 °C (the CJMA-7_fr-mod25_) and by 40% in the case of drying at 50 °C (the CJMA-7_fr-mod50_).

As was mentioned above, there are two changes in the membrane structure due to this modification. First, a thin film (about 4 microns) of PFSA form on the membrane upper side (Section 3.2). Second, the PFSA solution penetrates the membrane through large pores. It follows from the experimental observation that some amount of this solution passes through the membrane when it is poured on the upper side of the CJMA-7_fr_ membrane [44]. The electrostatic interactions between the $-SO_{3}^{-}$ fixed groups of the PFSI and $-N^{+}{\left(CH_{3}\right)}_{3}$ groups of the substrate membrane facilitate the penetration of the PFSI into macropores. When the PFSA solution is poured on the top of the membrane, it penetrates into it under the action of the gravitational and electrostatic forces, while the viscosity forces delay this penetration [68]. Over time, the solution fills the available pores and stops seeping through the membrane, so that some of it forms a film on the top side of the membrane. However, it is not likely that it fills them completely. Even if the PFSI solution occupies the entire macropore volume immediately after its introduction, after evaporation of the solvent (isopropyl alcohol), the PFSI volume decreases.

Let us evaluate the effect of the PFSI film on the membrane conductivity and diffusion permeability.

If we neglect the concentration dependence of the membrane diffusion permeability and conductivity, the integral coefficient of diffusion permeability ($P_{tot}$) and conductivity ($\kappa_{tot}$) of the modified bilayer membrane can be expressed as:

(9) $$P_{tot}=\left(d_{sub}+d_{film}\right){\left(\frac{d_{sub}}{P_{sub}}+\frac{d_{film}}{P_{film}}\right)}^{-1}\;\mathrm{and}\;\kappa_{tot}=\left(d_{sub}+d_{film}\right){\left(\frac{d_{sub}}{\kappa_{sub}}+\frac{d_{film}}{\kappa_{film}}\right)}^{-1}$$

where d is the thickness of one of the layers (the substrate or film); indices “*sub*” and “*film*” denotes the substrate (the CJMA-7_fr_ membrane) and a film, respectively.

As it can be seen from Equation (9), due to a very small value of the ratio $d_{film}/d_{sub}$ (equal to 4/174 = 0.023) in the case of a relatively thin modifying film, and relatively close values of $P_{sub}$ and $P_{tot}$, as well as $\kappa_{sub}$ and $\kappa_{tot}$, the resulting parameters of the bilayer membrane and substrate are quite close to each other. For example, in 1 M NaCl solution, the CJMA-7_fr_ membrane parameters are $P_{sub}$ = 27 × 10^−8^ cm^2^ s^−1^ and $\kappa_{sub}$ = 13.8 mS cm^−1^. For the PFSI in the same solution, $P_{film}$ = 8.6 × 10^−8^ cm^2^ s^−1^ and $\kappa_{film}$ ≈ 7.5 mS cm^−1^ (the data are taken for a MF-4SK membrane [69]). Calculations using Equation (9) yields $P_{tot}$ = 25.6 × 10^−8^ cm^2^ s^−1^ and $\kappa_{tot}$ = 13.5 mS cm^−1^. The corresponding difference in the transport number of Na^+^ (calculated using *P_tot_* and *κ_tot_*, Equation (12)) in the single layer membrane-substrate ($t_{Na^{+}sub}^{*}$= 0.054) and in the bilayer membrane ($t_{Na^{+}tot}^{*}$= 0.053) is quite small. However, the decrease in $P_{tot}$ and $\kappa_{tot}$, as well as in the sodium transport number, observed in the experiments [44] when passing from the CJMA-7_fr_ membrane to the CJMA-7_fr-mod25_ membrane is essentially greater (${\left(P_{tot}/P_{sub}\right)}_{\exp}\approx$ 3.2, ${\left(\kappa_{tot}/\kappa_{sub}\right)}_{\exp}\approx$ 2.5, ${\left(t_{Na^{+}tot}/t_{Na^{+}sub}\right)}_{\exp}\approx$ 0.053/0.03 = 1.8) than the above estimations show. Therefore, we can neglect the presence of a thin PFSI film on the modified membrane surface and take into account only the following two effects: (1) the penetration of the PFSI into the macropores of the substrate membrane, and (2) drying of the composite membrane at an elevated temperature.

(1) The fact that the liquid PFSI passes through the CJMA-7_fr_ membrane suggests that the PFSI material occupies only a part of the macropores, so that a space available for solution remains in the pore center (Figure 2c). The assumption that this material covers the pore walls follows from the presence of negatively charged fixed groups ($-SO_{3}^{-}$) in it. These groups can interact electrostatically with the positively charged fixed groups ($-N^{+}{\left(CH_{3}\right)}_{3}$) in the CJMA-7 membrane. Two oppositely charged functional groups can be quite close to each other, since PFSI is a liquid. They tend to neutralize their charges, however, the groups still have an attraction to cations and anions and tend to also associate with them [70]. It can be assumed that the $-SO_{3}^{-}$ fixed groups in thin nanometer layer of PFSI would be neutralized with the $-N^{+}{\left(CH_{3}\right)}_{3}$ groups of the CJMA-7 membrane located on the macropore walls. However, the PFSI material retains its functional groups at some distance from the pore walls. Even if a half of the functional groups of PFSI will be neutralized, the relative amount of the neutralized positively charged groups of the CJMA-7 membrane will be small compared to the total amount of this groups.

(2) When drying, the polymer loses water, which leads to a shrinkage of the membrane matrix and narrowing the pores. As a result, *f*_1_ increases (*f*_2_ decreases). In addition, the ion diffusion coefficients in the gel phase decrease (parameters ${\bar{D}}_{-}$ and ${\bar{D}}_{+}$). The higher the drying temperature, the greater changes in the above parameters (Table 1). The decrease in water content also leads to an increase in the gel exchange capacity $\bar{Q}$ (expressed in mmol cm^−3^) and a decrease in *α* (Table 1). As for the modelling of the contribution of the PFSI in the macropores, it is made by calculation of the $L_{+}$ and $L_{-}$ parameters (related to the intergel domains) using Equation (5). These parameters are calculated as for a homogeneous medium consisted of PFSI and an aqueous solution. In the case of 25 °C drying temperature, the diffusion coefficients $D_{+}$ and $D_{-}$ are taken 20% greater than those found for a strongly watered MF-4SK membrane [71] (with water content 36.5 mol H_2_O/mol functional groups, Table 2). The values of $D_{+}$ and $D_{-}$ are set lower in the case of 50 °C, in the same proportion as for the membrane-substrate. The concentrations of cations and anions are calculated using the Donnan equation, Equation (7), applied as for a cation-exchange membrane. However, the value of exchange capacity of the material filling the intergel spaces, $\bar{Q}$, is taken rather low, when assuming that PFSI fills 33% and 50% of the intergel space volume in the cases of 25 °C and 50 °C of the drying temperature, respectively. Since more water leaves the membrane when drying at 50 °C, the $\bar{Q}$ value for both membrane gel domains and intergel spaces is set greater at this temperature than at 25 °C. During drying, water molecules, which are more weakly bound to the ion-exchange material, leave the membrane first. Therefore, intergel spaces containing unbound water are the first to lose it. For this reason, the volume of intergel spaces decreases more than the volume of gel domains; therefore, the more the membrane is dried, the larger *f*_1_ and the smaller *f*_2_.

**Table 2:** Some characteristics of studied membrane.

Membrane	Fixed Groups	Thickness in 0.02 M NaCl, µm	Ion Exchange Capacity in Swollen State, mmol·g_wet_^−1^	Water Content, mol H_2_O/mol Fixed Groups	Density in Swollen State, g·cm^−3^
CJMA-7	Mainly $-N^{+}{\left(CH_{3}\right)}_{3}$	174 ± 10	0.75 ± 0.05	21.7 ± 1.0	1.13 ± 0.05
PFSI	$-SO_{3}^{-}$	-	0.87 ± 0.05 [71]	36.5 ± 1.0 [71]	^a^ 1.40 ± 0.15 [72]

^a^ Data for a Nafion material, an analogue of LF-4SK.

As Figure 1 shows, there is a good agreement between computational results and experimental data. We would like to show that each stage of membrane treatment can be described by a reasonable change of the membrane structural and kinetic parameters of the MH model. We discussed above only the direction of the parameters’ changes; their values were found by fitting the experimental data. However, the response of the parameter values to one or another method of membrane modification makes it possible to better understand what consequences this method entails. In particular, when we consider Equation (12), we see that, in order to increase the membrane permselectivity towards the counterion transport, we must decrease the value of P* and increase that of κ*: the membrane diffusion permeability, P*, is controlled by the transport of coions, and the conductivity, κ*, by the transport of counterions. Nevertheless, when changing some condition, usually both parameters vary in the same direction. For example, when decreasing water content (e.g., by drying the membrane at an elevated temperature), the mobilities of both counterion and coion decrease. With that, the effective mobility of coion decreases in a greater extent, since the pathway of this ion increases, as explained above. Therefore, this makes it possible to increase the membrane permselectivity, although at the expense of a decrease in the membrane conductivity. This is an example of the trade-off between membrane selectivity and permeability/conductivity, which is intensively discussed in the literature [37,42,73]. As it is shown in our paper, “clogging” the macropores with a polymer is a promising way to improve this trade-off towards higher selectivity without significant loss of conductivity.

Note that the penetration of the PFSI into the porous structure of the CJMA-7 anion-exchange membrane is facilitated by the interactions between the negatively charged $-SO_{3}^{-}$ fixed groups of the PFSI and positively charged $-N^{+}{\left(CH_{3}\right)}_{3}$ groups of the substrate membrane. The PFSI is literally drawn into the pores due to the attraction forces. Once this polymer is in a pore, some $-SO_{3}^{-}$ groups become neutralized, but others remain active. These active groups attract salt cations, which are co-ions for the CJMA-7 membrane, hence, they reduce the membrane permselectivity. We take into account this phenomenon by applying the Donnan equation (Equation (7)) to the intergel spaces partly filled with the PFSI and by introducing the Donnan coefficient *K_D_* = 0.1 for these spaces (Table 1). Therefore, we are aware that the fixed charge of the PFSI worsens the overall positive effect of the membrane modification with the PFSI. If we assume that all fixed groups of the PFSI are neutralized, i.e., the ion-exchange capacity, $\bar{Q}$, of the polymer within the pores is zero, then the model predicts that, with the same other parameters, the Na^+^ transport number in the membrane should be 0.025 instead of 0.03 (found experimentally at best). The question remains how to eliminate in practice the unwanted effect of the appearance of negatively charged fixed ions in the anion-exchange membrane pores when modifying it by a charged polyelectrolyte?

## 3. Experimental

The experimental results were obtained by Sarapulova et al. [44]. The authors modified a CJMA-7 (Hefei Chemjoy Polymer Materials Co. Ltd., Hefei, China) anion-exchange membrane (AEM) with a PFSI material. The modification process went through several stages. In order to reasonably describe the effect of each of the stages, we briefly present here the modification procedure.

### 3.1. Membrane and Modifier

The ion-exchange matrix of CJMA-7 membrane contains polyolefin functionalized with quaternary ammonium groups [74]. The matrix is crosslinked through the side chains [75]. This membrane is produced by the casting method and is reinforced with polyethylene terephthalate cloth by hot rolling.

A 7% isopropyl alcohol solution of a perfluorosulfonated ionomer (PFSI) LF-4SK (JSC Plastopolimer, St. Petersburg, Russia), which is a liquid precursor of the MF-4SK (JSC Plastopolimer, St. Petersburg, Russia) membrane, was used for the CJMA-7 membrane modification. This polymer is an analogue of the Nafion^TM^ material. Note that, when preparing a membrane using an PFSI solution by the solution-casting method, the obtained membrane exhibits a rather loose structure with a relatively high water content [76]. For this reason, for characterizing the PFSI material used in the modification [44], we have chosen the parameters of an MF-4SK membrane with a water content equal to 36.5 mol H_2_O/mol fixed groups reported by Berezina et al. [71].

Some of the relevant characteristics of the CJMA-7 membrane and the modifier are given in Table 2. The material of the reinforcing cloth is polyethylene terephthalate.

### 3.2. Membrane Modification

The CJMA-7 membrane was modified in several stages:

(1) a sample of a pristine CJMA-7 membrane (CJMA-7_pr_) was kept in a fridge for a year at a temperature of 4–6 °C in a 0.1 eq L^−1^ NaCl solution (CJMA-7_fr_); after that, the obtained membrane was divided in two samples;

(2) one of the samples was poured with a 7% PFSI solution (perfluorinated sulfonated polymer in isopropyl alcohol) and dried at room temperature for 24 h;

(3) then, the obtained membrane was divided into two more samples; both samples were dried for an extra 1 h: the first one at 25 °C (CJMA-7_fr-mod25_) and the second one at 50 °C (CJMA-7_fr-mod50_) in a drying oven.

Note that, when the PFSI solution was poured on the surface of the CJMA-7_fr_ membrane, it passed through the membrane so that some amount of this solution appeared on the opposite (down) membrane side. With that, a thin film with a thickness of about 4 μm was formed on the upper surface of the membrane [44].

Along with the CJMA-7_fr-mod25_ and CJMA-7_fr-mod50_ samples, the CJMA-7_pr_ and CJMA-7_fr_ samples were characterized. To equalize the conditions of preliminary preparation of the treated membranes, the CJMA-7_fr_ sample was kept in air at room temperature (25 °C) for 25 h. For the above four membranes (CJMA-7_pr_, CJMA-7_fr_, CJMA-7_fr-mod25_ and CJMA-7_fr-mod50_), the concentration dependencies of membrane conductivity, diffusion permeability and transport numbers were determined. The details of the experiments are described in [44].

**Figure 3:**
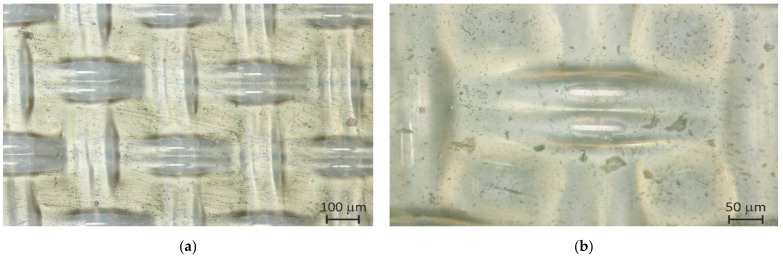
Optic images of the rougher surface of the pristine CJMA-7 membrane taken at different magnification: (**a**) 10× and (**b**) 20×.

**Figure 4:**
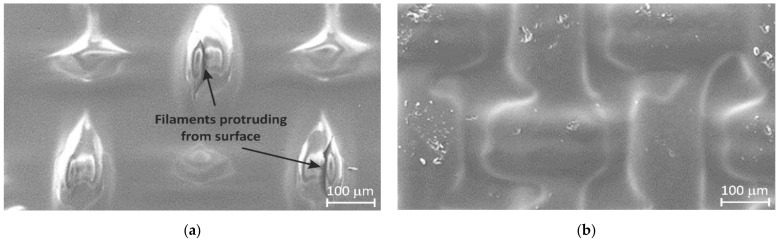
SEM images of the rougher surface of the pristine CJMA-7 membrane (**a**) and this surface after modification with a perfluorinated sulfonated ionomer LF-4SK (**b**).

The reinforcing cloth in the pristine CJMA-7 membrane is located closer to one of the membrane surfaces; this surface is rougher, while the other one is smoother. Figure 3 and Figure 4a show that parts of the filaments of the cloth protrude from the membrane surface. The modifier (a 7% PFSI solution) was applied on the rougher surface in order to coat the protrusions of the filaments. After modification, the membrane surface on which the modifier was applied became less rough; all the filaments were coated with a film of the modifier (Figure 3b).

The occurrence of relatively large macropores is evidenced by the fact that the PFSI solution passes through the membrane under the force of gravitation, as noted above. The most probable location of these pores is at the contacts between the filaments of the reinforcing cloth (fabricated from the polyethylene terephthalate) and the ion-exchange material. An experiment on drying the pristine CJMA-7 membrane is described in Ref. [44]. During drying, pores with a diameter of about 1 μm (detected from optical images) appear first at the crosshairs of the filaments, and then along the filaments. Taking into account that some crosshairs of the filaments are in direct contact with the bathing solution, it can be assumed that unhindered coion transport can occur through these large pores, some of which open into the external solution.

### 3.3. Membrane Characterization

The membrane conductivity ($\kappa^{*}$) was determined through the measurement of the membrane resistance by the differential method using a clip cell [77] at an alternating current frequency of 1 kHz. The integral diffusion permeability coefficient (P) was obtained using a two-compartment flow cell, where an electrolyte solution (1000 cm^3^) with a given concentration was circulated from one side of the membrane, and initially distilled water (100 cm^3^), from the other side [78]. The solute flux density ($J_{s}$) through the membrane was determined by the rate of the solute concentration growth in the stream of initially distilled water. The value of *P* was calculated as follows:

(10) $$P=\frac{J_{s}d_{m}}{C_{s}}$$

where $d_{m}$ is the membrane thickness and $C_{s}$ is the solute concentration in the stream of the feed solution. The solute concentration in the dilute solution (initially distilled water) was found via the conductivity measurements [44]. The differential ($P^{*}$) and integral permeability coefficients are linked as follows [59,79]

(11) $$P=\left(\int_{0}^{C_{s}}P^{*}(C)dC\right)/C_{s},\;P^{*}=P\left(1+\beta \right)$$

where $\beta ={d\log P/d\log C}_{s}$.

Thus, the value of $P^{*}$ can be obtained from the concentration dependence of *P* presented in bi-logarithmic coordinates (logP vs. $\log C_{s}$).

The coion transport number was calculated using the following equations [80]

(12) $$t_{A}^{*}=\frac{F^{2}}{2RT}\frac{P^{*}C_{s}}{\kappa^{*}t_{1}^{*}},\;t_{1}^{*}+t_{A}^{*}=1$$

where *F*, *R* and *T* are the Faraday and gas constants and the temperature, respectively. The condition $t_{1}^{*}+t_{A}^{*}=1$ allows one to find the transport numbers of both the counterion (index “1”) and coion (index “*A*”) using Equation (12).

## 4. Conclusions

The microheterogeneous model with some new amendments is applied to simulate the changes in membrane structure and related transport properties due to different modifications of an anion-exchange CJMA-7 membrane. The casting of a perfluorosulfonated ionomer solution on the CJMA-7 membrane surface resulted in (1) formation of a thin (4 μm) PFSI film on the surface of the membrane substrate, and (2) partial filling of the substrate macropores with the PFSI material.

In an experimental study made earlier [44], it was found that, after several stages of modification of the CJMA-7 membrane, the coion (Na^+^) transport number in the composite membrane equilibrated with 1 M NaCl solution decreased from 0.11 to 0.03.

The modeling shows that the effect of the additional film is negligible due to its thin thickness, and relatively close transport characteristics of the film and substrate. The main effect leading to a greater permselectivity of the modified membrane is partial blocking of macropores of the CJMA-7 membrane with the PFSI material.

The obtained results demonstrate that the method of filling the membrane macropores with a material that prevents coion transport is promising for a relatively easy modification, leading to an increase in the counterion permselectivity of ion-exchange membrane. This study proves the feasibility of this method. It is possible, however, that, in the case of other membranes and other modifiers, the increase in the counterion transport number would be greater than in the case reported in this paper.

## Figures and Tables

**Table 1:** Parameters of the MH model for the studied membrane samples in NaCl solution. PFSI-25 and PFSI-50 denote the PFSI material in the cases where the modified membrane was dried at 25 °C and 50 °C, respectively.

	*f*_1_	*f*_2_	${\bar{D}}_{-}$ × 10^6^, cm^2^ s^−1^	${\bar{D}}_{+}$ × 10^6^, cm^2^ s^−1^	$D_{-}$ × 10^6^, cm^2^ s^−1^	${\bar{D}}_{+}$ × 10^6^, cm^2^ s^−1^	α	$K_{D}$	$\bar{Q}$, mmol cm^−3^
CJMA-7_pr_	0.82	0.18	2.30	1.50	20.3	13.4	0.30	0.07	0.92
CJMA-7_fr_	0.84	0.16	2.25	0.65			0.25	0.05	0.94
CJMA-7_fr-mod25_	0.85	0.15	1.50	0.55	4.5	5.5	0.12	0.05	0.95
CJMA-7_fr-mod50_	0.90	0.10	1.45	0.50	4.3	5.2	0.10	0.05	1.0
PFSI-25	-	-	-	-	4.5	5.5	-	0.1	0.13
PFSI-50	-	-	-	-	4.3	5.2	-	0.1	0.18

## Supplementary Materials

The following are available online at https://www.mdpi.com/article/10.3390/ijms23042238/s1.
