# Supplementary figures and images for: Mathematical Description of the Increase in Selectivity of an Anion-Exchange Membrane Due to Its Modification with a Perfluorosulfonated Ionomer
Source: Int J Mol Sci. 2022 Feb 17;23(4):2238. doi: 10.3390/ijms23042238 (PMC8877549; doi:10.3390/ijms23042238)

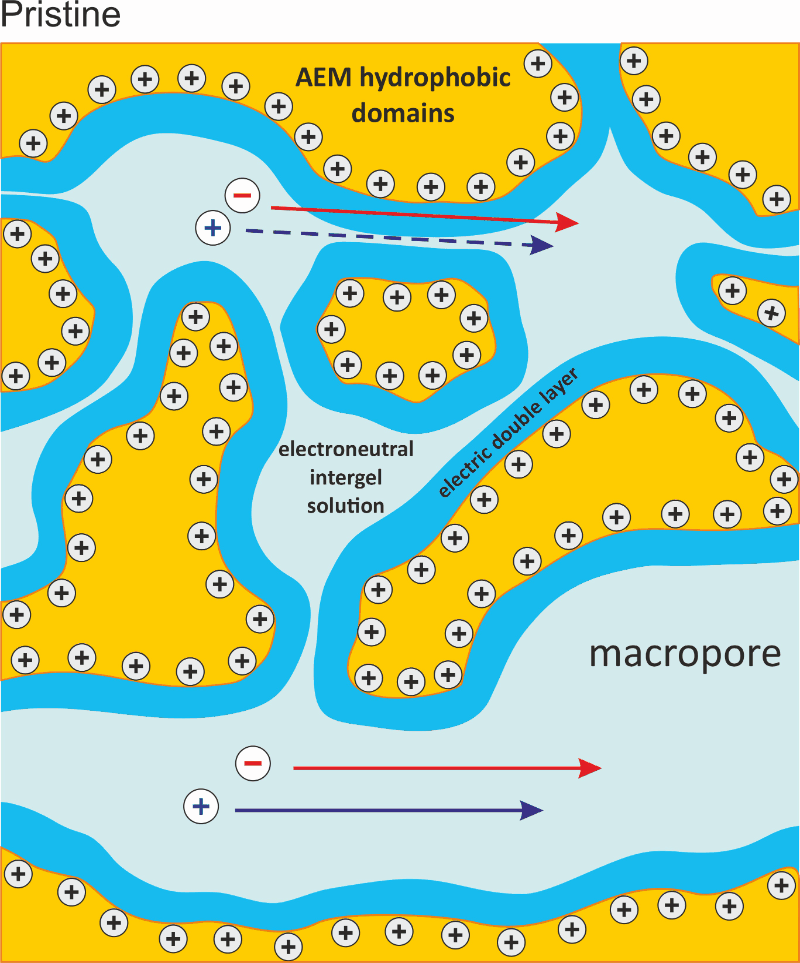

Supplement: Supplementary file 1 [file ijms-23-02238-s001.zip › ijms-1554810-supplementary.gif]
